# Routes to Chaos Induced by a Discontinuous Resetting Process in a Hybrid Spiking Neuron Model

**DOI:** 10.1038/s41598-017-18783-z

**Published:** 2018-01-10

**Authors:** Sou Nobukawa, Haruhiko Nishimura, Teruya Yamanishi

**Affiliations:** 1 Department of Computer Science, Chiba Institute of Technology, 2-17-1 Tsudanuma, Narashino, Chiba 275-0016 Japan; 2 Graduate School of Applied Informatics, University of Hyogo, 7-1-28 Chuo-ku, Kobe, Hyogo, 650-8588 Japan; 3 Department of Management Information Science, Fukui University of Technology, 3-6-1 Gakuen, Fukui, 910-8505 Japan

## Abstract

Several hybrid spiking neuron models combining continuous spike generation mechanisms and discontinuous resetting processes following spiking have been proposed. The Izhikevich neuron model, for example, can reproduce many spiking patterns. This model clearly possesses various types of bifurcations and routes to chaos under the effect of a state-dependent jump in the resetting process. In this study, we focus further on the relation between chaotic behaviour and the state-dependent jump, approaching the subject by comparing spiking neuron model versions with and without the resetting process. We first adopt a continuous two-dimensional spiking neuron model in which the orbit in the spiking state does not exhibit divergent behaviour. We then insert the resetting process into the model. An evaluation using the Lyapunov exponent with a saltation matrix and a characteristic multiplier of the Poincar’e map reveals that two types of chaotic behaviour (i.e. bursting chaotic spikes and near-period-two chaotic spikes) are induced by the resetting process. In addition, we confirm that this chaotic bursting state is generated from the periodic spiking state because of the slow- and fast-scale dynamics that arise when jumping to the hyperpolarization and depolarization regions, respectively.

## Introduction

Many types of neural coding (for e.g., rate, temporal, and population coding) are known to exist in brain/nerve system adaptive information processing^1^. Many recent studies on the mechanisms of memory and learning in neural systems have utilised spiking neuron models that can recreate these types of neural coding by describing the spiking activity of the membrane potential^1–5^.

The Hodgkin–Huxley neuron model^6^ is an important spiking neuron model that describes the dynamical evolution of the membrane potential and that of the gate variables associated with the K and Na ionic currents across the cellular membrane. This model comprises four equations involving several physiological parameters. Thus far, parameter values have been estimated for various types of neurons ranging from enormous squid axons to cortex neurons^6–9^. However, the systems that are obtained when the Hodgkin–Huxley neuron model is used to construct large-scale neural systems include many variables and parameters, approaching the scale of an actual brain neural network. Therefore, applicable analytical approaches are limited and the computational load of the numerical calculation becomes high.

Simplified models described by continuous differential equations have been proposed to overcome this difficulty. These models retain the minimum required bifurcations and spiking patterns (e.g. the FitzHugh–Nagumo neuron model^10,11^ and the Hindmarsh–Rose neuron model^12^).

Meanwhile, several hybrid spiking neuron models combining continuous spike generation mechanisms and discontinuous resetting processes after spiking have been proposed as simple transition schemes for membrane potentials between spiking and part of the hyperpolarization^13–16^. For example, the Izhikevich neuron model can reproduce nearly all spiking activities observed in actual neural systems by tuning a few parameters, including those relating to the resetting process^13,14^. Furthermore, in their use of non-linear integrate-and-fire models as hybrid spiking neuron models, Badel *et al*.^17^ proposed a possible method for estimating optimal parameter values using data obtained from a single neuron via intracellular voltage recording. They reported an extremely high fitting accuracy in reproducing spike patterns and were able to rapidly estimate parameters.

The hybrid spiking neuron model is a piecewise-smooth system in which the dynamics are switched according to the system’s state^18^. Saito *et al*. conducted chaos and bifurcation analysis and circuit implementation using the piecewise-constant and piecewise-linear systems as types of piecewise-smooth systems^19–23^. Tsubone *et al*.^23^ proposed a systematic method using an analytical approach to predict the parameter regions for the chaotic states in piecewise-constant systems. Meanwhile, Mitsubori and Saito^19^ and Nakano and Saito^20^ developed a systematic method for piecewise-linear systems. However, the analysis of chaos and bifurcation in piecewise-smooth systems such as the Izhikevich neuron model, which generally include non-linear terms, requires the evaluation of the Lyapunov exponents and characteristic multipliers against exhaustive parameter sets. Several indices considering the effect of the resetting process using the saltation matrix have been proposed^16,24–27^. Coombes *et al*.^16^ utilised this approach and conducts an analysis of the planar non-linear integrate-and-fire model in a large parameter region. They revealed that the parameter region for chaotic states is located at the boundary between burst firing and fast spiking. The Izhikevich neuron model clearly features various types of bifurcation and routes to chaos under the effect of the state-dependent jump in the resetting process^27–31^. However, neither the relation between the chaotic behaviours and the state-dependent jump nor the mechanism for inducing the chaotic states using the resetting process has been revealed.

Revealing this mechanism requires evaluating the influence of the state-dependent jump on the trajectory in a continuous system through a comparison of two systems: versions with and without the resetting process. The aforementioned Izhikevich neuron model cannot be applied for this purpose because of its divergent behaviour in the spiking state when the resetting process is removed. We previously introduced a preliminary approach to this issue based on a comparison of spiking neuron models such as the FitzHugh–Nagumo neuron model with and without the resetting process^32,33^. In actual neural systems, the system state transits from resting to spiking through both Hopf and saddle-node bifurcations^1,34^. However, the FitzHugh–Nagumo neuron model permits spiking activity only via Hopf bifurcation. A non-linear equation for the membrane recovery variable with a sigmoidal function has been reported to generate spiking activity via both types of bifurcation^34^.

In our approach, we first adopt a continuous two-dimensional (2D) spiking neuron model with a non-linear equation for the recovery variable in which the orbit in the spiking state does not exhibit divergent behaviour. We then add the resetting process to the model. Utilising a rigorous method to analyse the bifurcation and chaos in hybrid systems (i.e., using a characteristic multiplier of the Poincaré map and Lyapunov exponents with a saltation matrix), we evaluate several routes to chaos, which cannot be achieved in a hybridised FitzHugh–Nagumo neuron model^33^, by changing the resetting process parameters and comparing the structure of the attractor between the system versions with and without the resetting process.

## Model and Method

### Spiking Neuron Model with the Resetting Process

The FitzHugh–Nagumo neuron model^10,11^ is driven by 2D ordinary differential equations with the following form:

1 $$\dot{v}=v(a-v)(v-1)-u+I,$$

2 $$\dot{u}=bv-cu,$$

where *v* and *u* represent the membrane potential of a neuron and the membrane recovery variable, respectively. The parameter *a* determines the shape of a *v*-nullcline $(\dot{v}=0)$, while *b*/*c* and *c* exhibit the sensitivity of *u* and its time constant, respectively. In real-world neural systems, the system state transits from resting to spiking through both Hopf and saddle-node bifurcations^1,34^. However, the FitzHugh–Nagumo model only permits spiking activity via Hopf bifurcation. Spiking neuron models such as the Morris–Lecar neuron model^35^ can be used to generate spiking activity via both types of bifurcation. Such models apply the following non-linear equation for $\dot{u}$ with a sigmoidal function for *v* ^34,36^:

3 $$\dot{u}=\alpha (\frac{1}{1+\exp (-(v-\beta )/\varepsilon )}-u),$$

where *α* is the time constant for *u* and parameters *β* and *ε* determine the shape of the sigmoidal function. Instead of the linear equation given by Eq. (2), we use Eq. (3) with the parameters set to (*a*, *α*, *ε*) = (0.1, 0.1, 0.05) as the equation for $\dot{u}$.

For the continuous spiking neuron model above, we implement the resetting process given by the following equation:

4 $${\rm{if}}\,v\ge {v}_{{\rm{peak}}},{\rm{then}}\{\begin{array}{l}v\to {v}_{r},\\ u\to u+d,\end{array}$$

where *v*_*r*_ and *d* represent the after-spike reset values of the membrane potential *v* and the recovery variable *u*, respectively. In other words, we consider it to be a spike event if *v* reaches *v*_peak_. The state-dependent jump induced by Eq. (4) converges to a continuous trajectory under the condition *v*_*r*_ → *v*_peak_ and *d* → 0 in the case which *v*_peak_ is set to a maximum value of *v* for the orbit of the continuous spiking neuron model given by Eqs (1) and (3).

We numerically analysed this model in SUNDIALS, our non-linear differential/algebraic equation solver simulator, using the backward-differentiation formula method with Newton’s iteration^37^. As this function can detect intersection points between a trajectory and the user-defined manifolds, we utilise it to detect the points for *v* ≥ *v*_peak_. The time step for numerical integration is related to the time precision needed to detect intersection points. As the fixed width in this solver is not user-tunable, relative and absolute tolerances are set instead; we set these to sufficiently small values (10^−14^) to achieve sufficient numerical precision for the evaluation of a chaotic spiking activity and the detection of the intersection.

### Evaluation Indices

#### Lyapunov exponents

Lyapunov exponents with saltation matrices are utilised here to quantify the chaotic activity in the version of the spiking neuron model with the resetting process^25,26,28^. The evolution of the orthogonal vectors of perturbation **l**_*j*_ (*j* = 1, 2) for Eqs (1) and (3) in a system with a continuous trajectory in spike intervals between the *i*-th and (*i* + 1)-th times (*t*_*i*_ ≤ *t* ≤ *t*_*i*+1_) is described as follows:

5 $${\dot{{\rm{\Lambda }}}}^{i+1}(t,{t}_{i})=J(v,u,t){{\rm{\Lambda }}}^{i+1}(t,{t}_{i}),$$

where Λ^*i*+1^ is the matrix (**l**_1_, **l**_2_). The Jacobian matrix *J* for Eqs (1) and (3) is given as follows:

6 $$J=[\begin{array}{cc}-3{v}^{2}+2(a+1)v-a & -1\\ \frac{\alpha }{\varepsilon }\frac{1}{1+\exp ((-v-\beta )/\varepsilon )}(1-\frac{1}{1+\exp ((-v-\beta )/\varepsilon )}) & -\alpha \end{array}].$$

**l**_*j*_ is corrected by Gram–Schmidt orthonormalisation at intervals of 10^−3^ to maintain the orthogonality of **l**_*j*_:

7 $${{\bf{l}}}_{1}^{\perp }\to {{\bf{l}}}_{1},$$

8 $${{\bf{l}}}_{2}^{\perp }\to {{\bf{l}}}_{2}-\frac{{{\bf{l}}}_{1}\cdot {{\bf{l}}}_{2}}{|{{\bf{l}}}_{1}^{2}|}{{\bf{l}}}_{1}^{\perp },$$

9 $${{\rm{\Lambda }}}^{i+1}\to ({{\bf{l}}}_{1}^{\perp },{{\bf{l}}}_{2}^{\perp }\mathrm{).}$$

The saltation matrix at *t* = *t*_*i*_ is given by the following equation:

10 $${S}_{i}=[\begin{array}{cc}\frac{{\dot{v}}^{+}}{{\dot{v}}^{-}} & 0\\ \frac{{\dot{u}}^{+}-{\dot{u}}^{-}}{{\dot{v}}^{-}} & 1\end{array}],$$

where (*v* ^−^, *u* ^−^) and (*v* ^+^, *u* ^+^) represent the values of (*v*, *u*) before and after spiking, respectively. Λ^*k*^(*T* ^*k*+1^, *T* ^*k*^) (*k* = 0, 1, …, *N*−1) can be expressed as follows in case spikes arise in the range [*T* ^*k*^: *T* ^*k*+1^]^28^:

11 $${{\rm{\Lambda }}}^{k}({T}^{k+1},{T}^{k})={{\rm{\Lambda }}}_{i+1}({T}^{k+1},{t}_{i}){S}_{i}{{\rm{\Lambda }}}_{i}({t}_{i},{t}_{i-1})\cdots {S}_{2}{{\rm{\Lambda }}}_{2}({t}_{2},{t}_{1}){S}_{1}{{\rm{\Lambda }}}_{1}({t}_{1},{T}^{k}),$$

12 $${{\rm{\Lambda }}}^{k}({T}^{k},{T}^{k})=({{\bf{l}}}_{1}/|{{\bf{l}}}_{1}|,{{\bf{l}}}_{2}/|{{\bf{l}}}_{2}\mathrm{|).}$$

The initial perturbation Λ^0^(*T* ^0^, *T* ^0^) and the evolution period *τ* = *T* ^*k*+1^ − *T* ^*k*^ are set to unit matrix *E* and 0.1, respectively.

The Lyapunov spectrum *λ*_*j*_ is calculated as follows based on the norm of ${{\bf{l}}}_{j}^{k}$ (*j* = 1, 2) in Λ^*k*^ (*T* ^*k*+1^, *T* ^*k*^):

13 $${\lambda }_{j}=\frac{1}{N\tau }\sum _{k\mathrm{=0}}^{N-1}\,\mathrm{log}(|{{\bf{l}}}_{j}^{k}|),$$

where the evolution period for *λ*_*j*_ is set to *Nτ* = 10^5^ (*N* = 10^8^, *τ* = 10^−3^).

#### Poincaré map

We set a Poincaré section Ψ(*v* = *v*_peak_) to conduct a bifurcation analysis in a system with a state-dependent jump. The dynamics of the system behaviour on Ψ are given by the Poincaré map as follows:

14 $${u}_{i+l}={\psi }^{l}({u}_{i})\quad (l=1,2,\ldots ).$$

We evaluate here the profile of *ψ* ^*l*^ on a return map of *u*_*i*_ − *u*_*i*+*l*_. In practice, we solve Eqs (1),(3) and (4) against the initial values of (*v*_*r*_, *u*_0_), and obtain the *u* values that pass through Ψ at times *l* as *u* ^*l*^. The values (*u*_0_, *u*_*l*_) are plotted on a return map of *u*_*i*_ − *u*_*i*+*l*_.

#### Characteristic multiplier of the Poincaré map

The variational equations of Eqs (1) and (3) in a system with a continuous trajectory in spike intervals between the *i*-th and (*i* + 1)-th times (*t*_*i*_ ≤ *t* ≤ *t*_*i*+1_) are defined as follows:

15 $${\dot{{\rm{\Phi }}}}_{i+1}(t,{t}_{i})=J(v,u,t){{\rm{\Phi }}}_{i+1}(t,{t}_{i}),$$

where Φ indicates the state transition matrix. Φ(*t*, *t*_0_) can be expressed as follows for the case in which spikes arise in the range [*t* : *t*_0_]:

16 $${\rm{\Phi }}(t,{t}_{0})={{\rm{\Phi }}}_{i+1}(t,{t}_{i}){S}_{i}{{\rm{\Phi }}}_{i}({t}_{i},{t}_{i-1})\cdots {S}_{2}{{\rm{\Phi }}}_{2}({t}_{2},{t}_{1}){S}_{1}{{\rm{\Phi }}}_{1}({t}_{1},0).$$

The initial state transition Φ(*t*_0_, *t*_0_) is set here as the unit matrix *E*.

We calculate the characteristic multiplier of the solution for the *l* period (*u*_0_ = *ψ* ^*l*^ (*u*_0_)) as follows:

17 $${\mu }^{l}=\frac{\partial {\psi }^{l}}{\partial {u}_{0}},$$

where *u*_0_ indicates the initial value of orbit **x**_0_ = (*v*_*r*_, *u*_0_) at *t* = *t*_0_. The projection *P* and embedding *P* ^−1^ to and from the Poincaré section are defined as follows by local sections Π_0_ and Π_1_, respectively:

18 $$P\,:{{\rm{\Pi }}}_{0}\to {\rm{\Psi }}\quad {\bf{x}}\to w=u,$$

19 $${P}^{-1}\,:{\rm{\Psi }}\to {{\rm{\Pi }}}_{0}\quad u\to {\bf{x}}=(\begin{array}{l}{v}_{r}\\ u\end{array}),$$

where *w* indicates the local coordinate on Ψ. According to the literature^27^, local sections Π_0_ and Π_1_ are set as follows to solve Eq. (17):

20 $${{\rm{\Pi }}}_{0}=\{{\bf{x}}=(v,u)\in {R}^{2}|{q}_{0}(v,u)=v-{v}_{r}=\mathrm{0\},}$$

21 $${{\rm{\Pi }}}_{1}=\{{\bf{x}}=(v,u)\in {R}^{2}|{q}_{1}(v,u)=v-c=\mathrm{0\},}$$

where *q*_0_(*v*, *u*) and *q*_1_(*v*, *u*) are the scalar functions used to determine the local sections. Eq. (17) can be developed as follows to utilise the aforementioned sets (see the detailed derivation from Eq. (22) to Eq. (23) in ref.^27^):

22 $$\begin{array}{rcl}{\mu }^{l} & = & \frac{\partial {\psi }^{l}}{\partial {{\bf{u}}}_{{\bf{0}}}}=\frac{\partial P}{\partial {\bf{x}}}\frac{\partial {\psi }^{l}}{\partial {{\bf{x}}}_{0}}\frac{\partial {P}^{-1}}{\partial w}\end{array}$$

23 $$=(\begin{array}{ll}0 & 1\end{array})(\begin{array}{cc}0 & 0\\ -\dot{v}/\dot{u} & 1\end{array}){\rm{\Phi }}({t}_{l},{t}_{0})(\begin{array}{c}0\\ 1\end{array})\mathrm{.}$$

|*μ* ^*l*^ < 1|, *μ* ^*l*^ = −1 and *μ* ^*l*^ = 1 represent the stable condition, period-doubling bifurcation and tangent bifurcation, respectively.

## Results

### Bifurcation in a Continuous 2D Spiking Neuron Model

**Figure 1:**
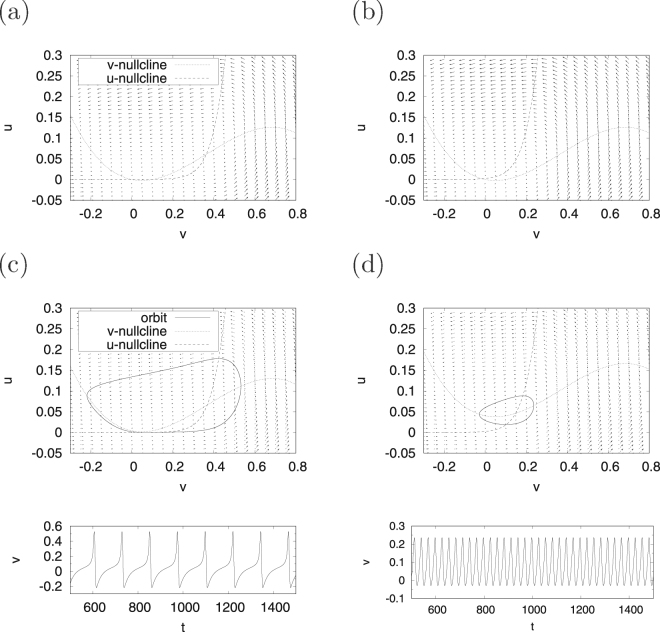
System behaviour in the continuous spiking neuron model. Orbit of (*v*, *u*) under the condition of no application of a direct current *I* = 0 in regions #1 (**a**) and #2 (**b**); the orbit of (*v*, *u*) (upper) and the time series of *v* (lower) under the condition with application of a direct current in regions #1 (*I* = 0.004) (**c**) and #2 (*I* = 0.04) (**d**). (*a* = 0.1, *α* = 0.1, *ε* = 0.05; *β* = 0.5 (region #1), *β* = 0.3 (region #2)).

First, we demonstrate the system behaviour of the continuous spiking neuron model given by Eqs (1) and (3) for the cases of *β* = 0.5 and *β* = 0.3, which are hereinafter called regions #1 and #2, respectively. For region #1, Fig. 1(a) shows the *v*-nullcline $(\dot{v}=0)$, *u*-nullcline $(\dot{u}=0)$ and vector field of $(\dot{v},\dot{u})$ in the case *I* = 0; these are denoted by a dotted line, a dashed line, and arrows, respectively. In this region, the fixed points (i.e. the points at which the *v*-nullcline intersects with the *u*-nullcline) are located at (*v*, *u*) ≈ (0, 0), (0.10, 0), (0.35, 0.06). The system trajectories are attracted to the stable fixed point ((*v*, *u*) ≈ (0, 0)). The other intersection points are unstable fixed points. In the *I* = 0.004 case (upper panel of Fig. 1(c)), the orbit indicated by a solid line exhibits a limit cycle along the vector field because of the effects of the destruction of the pair of stable and unstable fixed points. Next, as shown in the lower panel of Fig. 1(c), a spiking activity appears in the time series of *v*(*t*). In region #2, the fixed point is located at (*v*, *u*) ≈ (0, 0), and, as the point is stable, the system trajectories are attracted to it (Fig. 1(b)). Meanwhile, in the *I* = 0.04 case, a limit cycle emerges as a result of the destabilisation of the fixed point (Fig. 1(d)).

**Figure 2:**
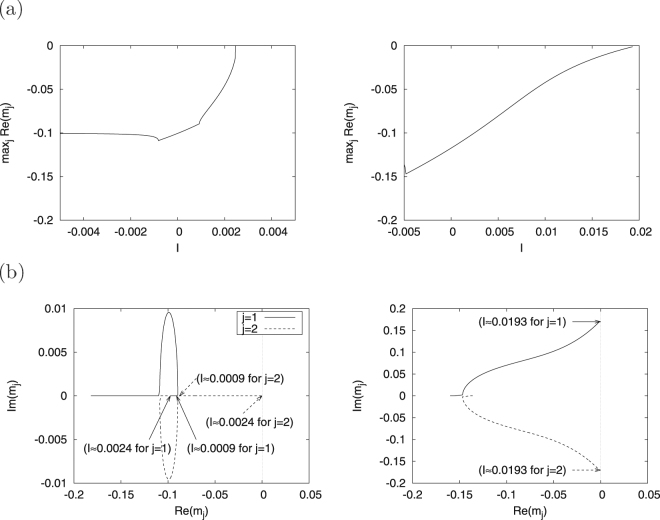
Dependence of the maximum real part of eigenvalue *m*_*j*_ (*j* = 1, 2) on parameter *I* (**a**) and dependence of *m*_*j*_ on parameter *I* (**b**) in regions #1 (left) and #2 (right). (*a* = 0.1, *α* = 0.1, *ε* = 0.05; *β* = 0.5 (region #1), *β* = 0.3 (region #2)).

We evaluate the eigenvalues *m*_*j*_ (*j* = 1, 2) of *J* around the stable fixed point ((*v*, *u*) ≈ (0, 0)) to specify the bifurcation against *I*. Figure 2(a) shows the dependence of the maximum real part of eigenvalue ${{\rm{\max }}}_{j}{\rm{Re}}({m}_{j})$ on *I*. The fixed point is stable $({{\rm{\max }}}_{j}{\rm{Re}}({m}_{j}\mathrm{) < 0})$ in $-0.005\le I\lesssim 0.0024$, but disappears when $I\gtrsim 0.0024$. Furthermore, the limit cycle can be interpreted to be generated through saddle-node bifurcation becasue the complex values of *m*_*j*_, *m*_1_ and *m*_2_ are real numbers in $0.0009\lesssim I\lesssim 0.0024$ and *m*_2_ reaches Re(*l*_2_) = 0 at *I* ≈ 0.0024, as shown in the left panel of Fig. 2(b). For region #2, the right panel of Fig. 2(a) shows the dependence of ${{\rm{\max }}}_{j}{\rm{Re}}({m}_{j})$ on *I*. In this region, the fixed point is stable in $-0.005\le I\lesssim 0.0193$ but becomes unstable when $I\gtrsim 0.0193$ because the complex values of *m*_*j*_, *m*_1_ and *m*_2_ are complex conjugates and pass through a real axis at *I* ≈ 0.0193 (right panel of Fig. 2(b)). Therefore, this bifurcation is classified as Hopf bifurcation.

### Bifurcation and Chaos in the Spiking Neuron Model with the Resetting Process

**Figure 3:**
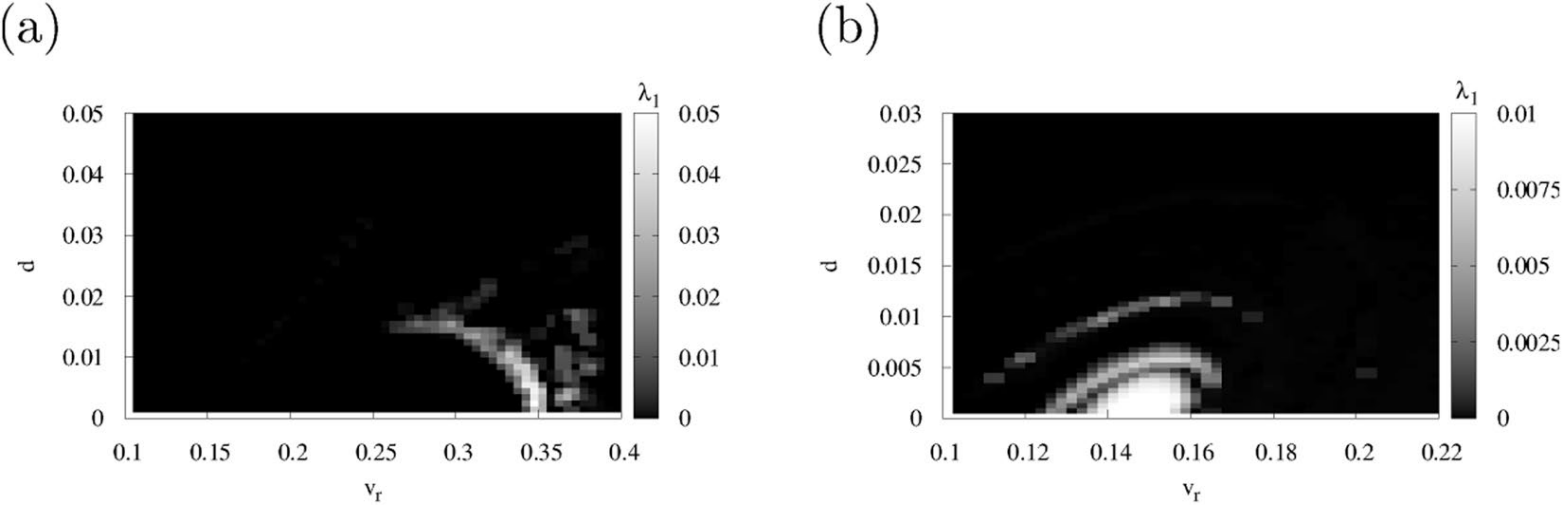
Maximum Lyapunov exponent *λ*_1_ as a function of *v*_*r*_ and *d* around the chaotic region (*a* = 0.1, *α* = 0.1, *ε* = 0.05). (**a**) Region #1 (*β* = 0.5, *I* = 0.004, *v*_peak_ = 0.4). (**b**) Region #2 (*β* = 0.3, *I* = 0.04, *v*_peak_ = 0.225).

We evaluate the system’s behaviour in the version of the spiking neuron model with the resetting process given by Eqs (1),(3) and (4). Figure 3 shows the dependence of the maximum Lyapunov exponent of *λ*_1_ on parameters *v*_*r*_ and *d* in regions #1 (a) and #2 (b), respectively. The parameters for Eqs. (1) and (3) are set to *a* = 0.1, *α* = 0.1, *ε* = 0.05; *β* = 0.5, *I* = 0.004 (region #1), *β* = 0.3, *I* = 0.04 (region #2) in the same manner as in the cases shown in Fig. 1(c) and (d). Parameter *v*_peak_ in Eq. (4) is set to 0.4 and 0.225 in regions #1 and #2, respectively. The chaotic state is induced by the resetting process in $0.25\lesssim {v}_{r}\lesssim 0.4,0\lesssim d\lesssim 0.025$ (region #1) and $0.12\lesssim {v}_{r}\lesssim 0.17,0\lesssim d\lesssim 0.01$ (region #2).

**Figure 4:**
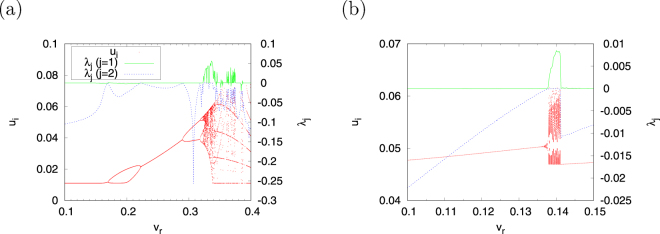
Bifurcation diagram and Lyapunov exponents *λ*_*j*_ (*j* = 1, 2) as functions of *v*_*r*_ around the chaotic region (*a* = 0.1, *α* = 0.1, *ε* = 0.05). (**a**) Region #1 (*β* = 0.5, *I* = 0.004, *v*_peak_ = 0.4, *d* = 0.01). (**b**) Region #2 (*β* = 0.3, *I* = 0.04, *v*_peak_ = 0.225, *d* = 0.01).

Figure 4 shows the dependence of the bifurcation diagrams of *u*_*i*_ and *λ*_*j*_ (*j* = 1, 2) on the *v*_*r*_ parameter in regions #1 and #2 with the value of parameter *d* fixed at *d* = 0.01 in Fig. 3. This result shows the chaotic states, which exhibit an irregular behaviour of *u*_*i*_, and the Lyapunov exponents *λ*_1_ > 0, *λ*_2_ = 0 in region #1 observed in the parameter set for ($0.322\lesssim {v}_{r}\lesssim 0.388$). We evaluate the bifurcation against the chaotic region. The period-doubling and tangent bifurcations arise at *v*_*r*_ ≈ 0.288 (*l* = 1), 0.318 (*l* = 2), 0.322 (*l* = 4), … and *v*_*r*_ ≈ 0.388 (*l* = 5). Therefore, the aforementioned chaotic region is produced by period-doubling bifurcation in the positive direction of *v*_*r*_ and by tangent bifurcation in the negative direction. The chaotic state (*λ*_1_ > 0, *λ*_2_ = 0) in region #2 is observed in $0.136\lesssim {v}_{r}\lesssim 0.141$ (Fig. 4(b)). Tangent bifurcation arises at both sides of this chaotic region (*v*_*r*_ ≈ 0.136 (*l* = 2), 0.141 (*l* = 1)). In other words, this chaotic region is induced by tangent bifurcation.

**Figure 5:**
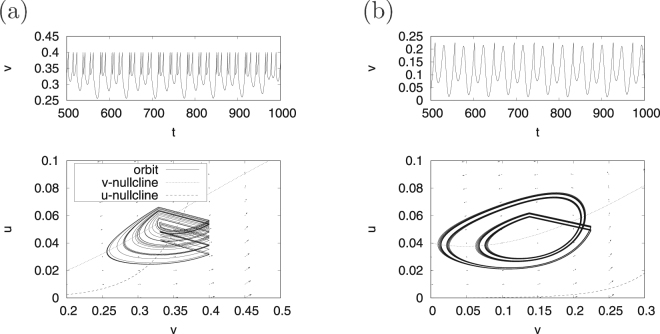
Chaotic time series of *v*(*t*) (upper) and the orbit of (*v*, *u*) (lower) in the spiking neuron model with a state-dependent jump in regions #1 (**a**) and #2 (**b**). (*a* = 0.1, *α* = 0.1, *ε* = 0.05. Region #1: *β* = 0.5, *I* = 0.004, *v*_peak_ = 0.4, *v*_*r*_ = 0.33 and *d* = 0.01; and region #2: *β* = 0.3, *I* = 0.04, *v*_peak_ = 0.225, *v*_*r*_ = 0.14 and *d* = 0.01).

**Figure 6:**
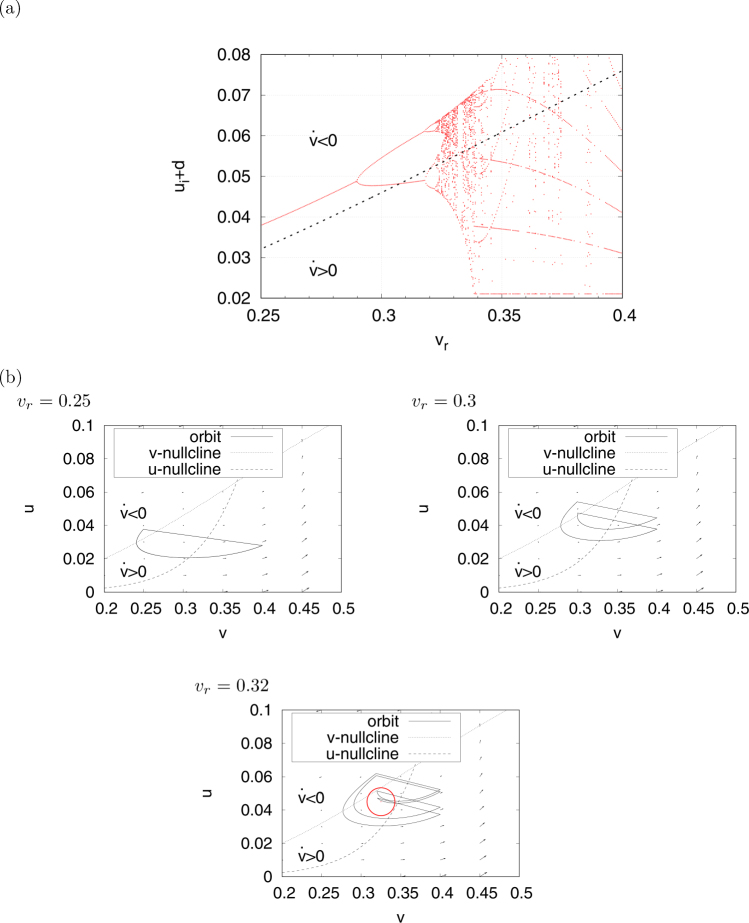
Transition scheme from spiking to bursting against changing *v*_*r*_ in region #1. (**a**) Bifurcation diagram of *u*_*i*_ + *d* (the dotted line indicates *v*-nullcline) and (**b**) orbits (*v*, *u*). The parameter values are similar to those in Figs 4 and 5 (*a* = 0.1, *α* = 0.1, *ε* = 0.05, *β* = 0.5, *I* = 0.004, *v*_peak_ = 0.4, *d* = 0.01). The red circle in the graph for *v*_*r*_ = 0.32 indicates the region, where (*v*, *u*) jumps in $\dot{v} > 0$.

The upper panel of Fig. 5(a) shows the time series of *v*(*t*) as an example of the chaotic spiking pattern in region #1 (*v*_*r*_ = 0.33, *d* = 0.01). In this spiking pattern, *v*(*t*) exhibits two types of behaviours after the resetting process (spike). In one case, *v*(*t*) enters the hyperpolarization mode and decreases to *v*(*t*) ≈ 0.25. In the other case, *v*(*t*) increases to *v*_peak_ but does not do so through hyperpolarization. This spike repeats several times. In the former behaviour, (*v*, *u*) jumps in the region of $\dot{v} < 0$ through the *v*-nullcline in the *v*-*u* phase plane, as shown in the lower panel in Fig. 5(a). Meanwhile, in the latter behaviour, (*v*, *u*) jumps in the region of $\dot{v} > 0$ but not do so through the *v*-nullcline via the resetting process. This spiking pattern is called a burst and is observed in actual neural systems. Figure 6 shows the bifurcation diagram after the value of *u*_*i*_ + *d* is reset (a) and typical orbits at *v*_*r*_ = 0.25, 0.3, 0.32 (b) for the transition scheme from spike to burst against changing *v*_*r*_. This result implies that (*v*,*u*) jumps in the region of $\dot{v} < 0$ at ${v}_{r}\lessapprox \,0.31$ (see typical examples *v*_*r*_ = 0.25, 0.3 in Fig. 6(b)). Meanwhile, at ${v}_{r}\gtrsim 0.31$, (*v*,*u*) jumps in the region of $\dot{v} > 0$ as well as in the region of $\dot{v} < 0$ (red circle in the *v*_*r*_ = 0.32 graph in Fig. 6(b)). The upper and lower panels of Fig. 5(b) show examples of the chaotic time series of *v* (*t*) and the behaviour of (*v*, *u*) in the *v*-*u* phase plane in region #2 (*v*_*r*_ = 0.14, *d* = 0.01). In this case, (*v*, *u*) always jumps in the region $\dot{v}\mathrm{ > 0}$ and the orbit exhibits a near-period-2 chaotic behaviour. We can also confirm the aforementioned mechanisms for generating a chaotic bursting behaviour and a near-period-2 chaotic behaviour in the Izhikevich neuron model^29,31^.

**Figure 7:**
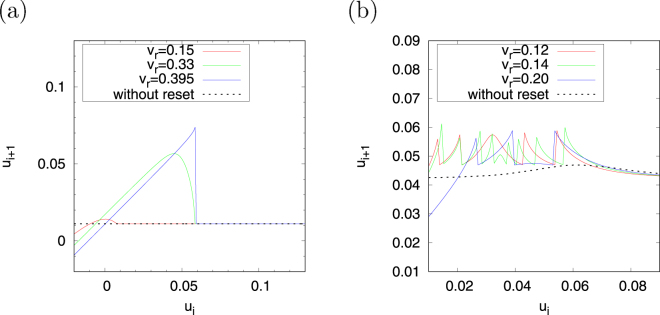
Dependence of the return map *u*_*i*+1_ = *ψ*(*u*_*i*_) on parameter *v*_*r*_ in regions #1 (**a**) and #2 (**b**). (*a* = 0.1, *α* = 0.1, *ε* = 0.05, *d* = 0.01. Region #1: *β* = 0.5, *I* = 0.004 and *v*_peak_ = 0.4; and region #2: *β* = 0.3, *I* = 0.04 and *v*_peak_ = 0.225).

Next, we investigate the dependence of the return map on *v*_*r*_. For region #1, Fig. 7(a) shows *ψ*(*u*_*i*_) on the *u*_*i*+1_ − *u*_*i*_ return map in cases with the resetting process, in which *v*_*r*_ = 0.15, *v*_*r*_ = 0.33 (corresponding to the value of *v*_*r*_ in Fig. 5(a)) and *v*_*r*_ = 0.395, and in the case without the resetting process. In the case without the resetting process, which is indicated by the dotted black line, *ψ*(*u*_*i*_) exhibits a nearly constant value (≈0.01). Meanwhile, in the cases in which the resetting process is applied the stretching and folding structure with piecewise nearly linear maps (*ψ*(*u*_*i*_) ≈ *u*_*i*_ + 0.01 if $-0.02\lesssim {u}_{i}\lesssim 0.06$ and *ψ*(*u*_*i*_) ≈ 0.01 if $0.06\lesssim {u}_{i}\lesssim 0.1$) in the *v*_*r*_ = 0.395 case is indicated by the solid blue line. In the case of *v*_*r*_ = 0.33, in which the distance of the jump in the *v* − *u* phase plane becomes larger than *v*_*r*_ = 0.395 as a result of separation from *v*_peak_ = 0.4, a stretching and folding structure with non-linearity emerges at *u*_*i*_ ≈ 0.04. The chaotic spiking activity observed in Fig. 5(a) can be considered to be induced by this structure. The stretching and folding structure then fades as *v*_*r*_ further decreases, as in the *v*_*r*_ = 0.15 case indicated by the solid red line. For region #2, Fig. 7(b) shows *ψ* (*u*_*i*_) on the *u*_*i*+1_ − *u*_*i*_ return map in the cases with the resetting process, in which *v*_*r*_ = 0.12, *v*_*r*_ = 0.14 (corresponding to the value of *v*_*r*_ in Fig. 5(b)), and *v*_*r*_ = 0.20, and in the case without the resetting process. *ψ* (*u*_*i*_) is a nearly constant value in the case without the resetting process (≈0.03), which is indicated by the dotted black line, as well as for region #1. However, the stretching and folding structure arises as an effect of the resetting process (*v*_*r*_ = 0.20 case, indicated by the solid blue line). Meanwhile, the frequency of the stretching and folding structure increases in the case in which the jump distance is larger than *v*_*r*_ = 0.20 (*v*_*r*_ = 0.14 case indicated by the solid green line. The near-period-2 chaotic spiking activity can be interpreted as being produced by this structure. This frequency decreases as *v*_*r*_ further decreases (*v*_*r*_ = 0.12 case indicated by the solid red line).

**Figure 8:**
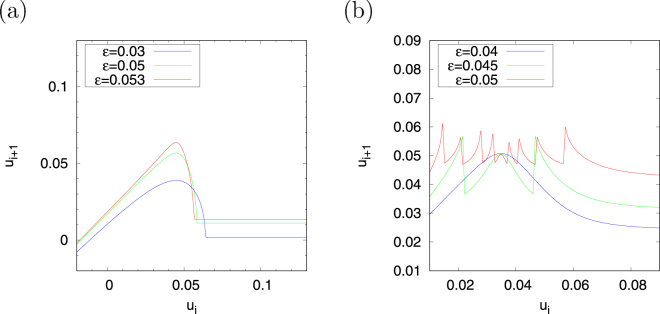
Dependence of the return map *u*_*i*+1_ = *ψ*(*u*_*i*_) on parameter *ε* in regions #1 (**a**) and #2 (**b**). (*a* = 0.1, *α* = 0.1, *d* = 0.01. Region #1: *β* = 0.5, *I* = 0.004, *v*_peak_ = 0.4 and *v*_*r*_ = 0.33; and region #2: *β* = 0.3, *I* = 0.04, *v*_peak_ = 0.225 and *v*_*r*_ = 0.14).

Fixing *v*_*r*_ at 0.33 in region #1 and *v*_*r*_ at 0.14 in region #2, we evaluate the dependence of *ψ*(*u*_*i*_) on the *u*_*i*+1_ − *u*_*i*_ return map on parameter *ε* (Fig. 8). The stretching and folding structure with piecewise nearly linear maps appears, as shown in *ε* = 0.053, in the case in which *ε* increases in region #1 (corresponding to the shape of the *u*-nullcline approaching the step function). A stretching and folding structure with non-linearity emerges at *u*_*i*_ ≈ 0.04 when the *ε* values decrease, such as at *ε* = 0.05, 0.03. In region #2, nine folding structures appear at *ε* = 0.05. The frequency of folding in this case decreases as the value of *ε* decreases (see *ε* = 0.045, 0.04).

## Discussion and Conclusion

In this paper, we applied the resetting process to a continuous 2D spiking neuron model with a sigmoidal nullcline structure to reveal the mechanisms for the emergence of chaotic states in a hybrid spiking neuron model. We also evaluated the bifurcation and routes to chaos against two types of spikes generated by the parameter sets for saddle-node bifurcation (region #1) and spikes for Hopf bifurcation (region #2) by changing the value of the resetting parameter. Through an evaluation using the Lyapunov exponent with a saltation matrix and the index for the fixed-point stability on a Poincaré section, we demonstrated that two types of chaotic behaviour are induced by the resetting process.

A chaotic state with bursting characteristics emerged in region #1 through tangent bifurcation, with a jump distance that increased as *v*_*r*_ decreased. This chaotic state moved to the periodic state through period-doubling bifurcation as the distance increased even further. We investigated the dependence of the return map on the resetting parameter and compared the models with and without the resetting process. Consequently, we found that the non-linear stretching and folding structure of the attractor is induced by the resetting process. This structure is also affected by the shape of the sigmoidal function of the *u*-nullcline. We also confirmed that the bursting chaotic states emerged according to the adjustment of this structure’s non-linearity.

Homoclinic orbits coexisting with fast and slow dynamics are needed to reproduce bursting^38^. For example, using the Hindmarsh–Rose neuron model as a continuous three-dimensional spiking neuron model can reproduce the bursting by fast dynamics of the membrane potential and recovery variable and the slow dynamics of the bursting variable^12^. Meanwhile, in hybrid spiking neuron models (Fig. 6) bursting can be reproduced using 2D systems because the hyperpolarization after resetting to $\dot{v} < 0$ and the depolarization in the inter-burst term after resetting to $\dot{v} > 0$ can play the roles of slow and fast dynamics, respectively. The analysis of the Hindmarsh–Rose neuron model indicated that a unimodal peak on the return map of the Poincaré section existed in chaotic bursting^39–41^. This structure, which was also confirmed in region #1 of our model (Fig. 7(a)), contributes to the generation of chaotic bursting. Investigations of this chaotic bursting by inter-spike intervals (ISI) have previously been described in the literature^42,43^. Gu^43^ reported that the return map of the ISI exhibited a unimodal peak in chaotic bursting, with a peak value that decreased with the approach of chaotic spiking in a physiological experiment involving a neural pacemaker. Meanwhile, Innocenti *et al*.^42^ demonstrated that this return map has two peaks in chaotic bursting and that these peaks merged with the approach of chaotic spiking in the Hindmarsh–Rose neuron model. The patterns of the ISI prior to the last-minute hyperpolarization might be interpreted as reflecting the number of peaks in the return map. In our case, we confirmed that the trend in our result is consistent with that of Gu (Additional Information)^43^.

A chaotic state with a near-period-2 behaviour emerged in region #2 through tangent bifurcation as the jump distance increased. As the distance was increased even further, this chaotic state moved to a periodic state through tangent bifurcation. Evaluation of the return map showed that this chaotic state emerged from the non-linear stretching and folding structure of the attractor induced by the resetting process. The folding frequency increased as the jump distance increased; also, this frequency decreased as the value of parameter *ε* decreased.

In conclusion, the resetting process provides and enhances non-linear effects in attractors, a result that cannot be achieved in continuous spiking neuron models of less than two dimensions. Chaotic states tend to arise when a state-dependent jump exists with an appropriate distance. The two types of chaotic behaviour and bifurcation mentioned above can also be observed in the widely used Izhikevich neuron model^14,29,31^. Therefore, the effects induced by the resetting process revealed in this study might be utilised to generate various chaotic spiking patterns.

Further research based on this study should be undertaken to classify the types of transition from chaotic bursting to spiking and to evaluate the bifurcation and chaos in neural networks composed of hybrid spiking neurons.

**Figure 9:**
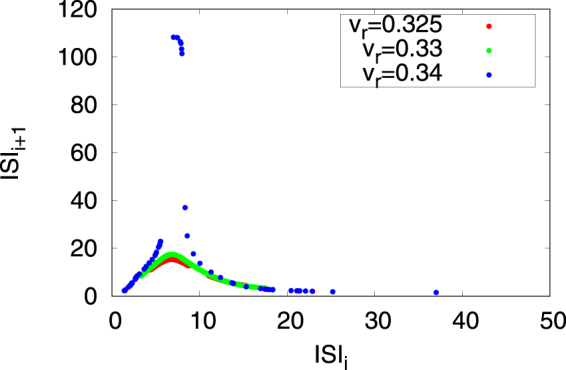
Return map of the inter-spike intervals (ISI) of chaotic bursting in region #1. (*a* = 0.1, *α* = 0.1, *ε* = 0.05, *β* = 0.5, *I* = 0.004, *v*_peak_ = 0.4, *d* = 0.01).

Figure 9 shows the inter-spike intervals (ISI) *ISI*_*i*_ = *t*_*i*+1_ − *t*_*i*_, where *t*_*i*_ indicates the spike time (*i* = 0, 1, 2,…) in the cases with the parameter settings for chaotic activity (*v*_*r*_ = 0.325, 0.33, 0.34) in region #1. As a result, the return map of the ISI exhibits a unimodal peak in chaotic bursting, and its peak value decreases with the approach of the parameter region of periodic bursting and spiking (Fig. 6).
